# A Bivariate Mapping Tutorial for Cancer Control Resource Allocation Decisions and Interventions

**DOI:** 10.5888/pcd17.190254

**Published:** 2020-01-02

**Authors:** Claire Biesecker, Whitney E. Zahnd, Heather M. Brandt, Swann Arp Adams, Jan M. Eberth

**Affiliations:** 1 Rural and Minority Health Research Center, Arnold School of Public Health, University of South Carolina, Columbia, South Carolina; 2 Department of Health Promotion, Education, and Behavior, Arnold School of Public Health, University of South Carolina, Columbia, South Carolina; 3 Department of Epidemiology and Biostatistics, Arnold School of Public Health, University of South Carolina, Columbia, South Carolina; 4 College of Nursing, University of South Carolina, Columbia, South Carolina

## Abstract

Bivariate choropleth mapping is a straightforward but underused method for displaying geographic health information to use in public health decision making. Previous studies have recommended this approach for state comprehensive cancer control planning and similar efforts. In this method, 2 area-level variables of interest are mapped simultaneously, often as overlapping quantiles or by using other classification methods. Variables to be mapped may include area-level (eg, county level) measures of disease burden, health care use, access to health care services, and sociodemographic characteristics. We demonstrate how geographic information systems software, specifically ArcGIS, can be used to develop bivariate choropleth maps to inform resource allocation and public health interventions. We used 2 types of county-level public health data: South Carolina’s Behavioral Risk Factor Surveillance System estimates of ever having received cervical cancer screening, and a measure of availability of cervical cancer screening providers that are part of South Carolina’s Breast and Cervical Cancer Early Detection Program. Identification of counties with low screening rates and low access to care may help inform where additional resources should be allocated to improve access and subsequently improve screening rates. Similarly, identifying counties with low screening rates and high access to care may help inform where educational and behavioral interventions should be targeted to improve screening in areas of high access.

**Summary**

**What is already known about this subject?**

Bivariate choropleth mapping is a way of displaying categories of values for 2 variables simultaneously on a map. It may be useful for public health decision making, such as cancer control planning.

**What is added by this report?**

This report provides a step-by-step guide for developing a bivariate choropleth map using ArcGIS software, and describes how to interpret the resultant map.

**What are the implications for public health practice?**

The development and use of these maps can guide allocation of resources and targeting of interventions to areas of greatest need.

## GIS Mapping to Inform Cancer Prevention and Control Efforts

Mapping by using geographic information systems (GIS) has been used extensively to inform resource allocation and to evaluate access to health care services, including cancer screening (1–4). By using area-level data, one can map estimates or rates of cancer risk factors; cancer screening use, incidence, and mortality; and access to cancer prevention, screening, and treatment services. Understanding the geographic distribution of cancer-related risk factors and outcomes can inform interventions by identifying areas of greatest burden of disease and greatest scarcity of services (5).

Bivariate mapping has been proposed as an effective GIS-based approach for disease surveillance and state-level public health programming broadly and cancer control planning specifically (6–9). This approach uses choropleth mapping (ie, a thematic map where areas are colored to represent data values) to display 2 variables simultaneously among geographic units such as states or counties by creating “n × n” groupings where values of both variables intersect. State-level studies have used bivariate mapping to examine lung cancer mortality relative to access to lung cancer screening, racial disparities in 2 types of cancer screening, late-stage rates of 2 cancers, and other cancer-related data (6,10–13). Overall, bivariate mapping is underused in cancer control, especially at the sub-state level (eg, counties). Applying this mapping approach within states may inform resource allocation and program planning. Our objective was to describe potential applications of bivariate mapping and provide step-by-step guidance for its implementation using GIS software to inform cancer prevention and control.

## Uses of Bivariate Choropleth Mapping in Cancer Prevention and Control

Data can be mapped in several ways for cancer prevention and control purposes by using public health surveillance, programming, policy, and other data (Table 1). Such data may include availability of public health programming locations and of screening and/or safety net providers, state-level policies, population-level rates of screening use, cancer incidence, staging rates, and/or mortality (7,10,14,15). Such data are generally accessible to state and local public health departments from sources like the Behavioral Risk Factor Surveillance System (BRFSS), cancer registries, Robert Wood Johnson Foundation County Health Rankings, and vital statistics systems. Bivariate mapping will be most appropriately implemented using state- and county-level data. Because of limitations in the availability of more geographically granular data and the subsequent challenges of calculating stable rates from sparse data, bivariate mapping at geographically smaller units, such as ZIP codes or census tracts, is not recommended. Bivariate maps are either 2×2 or 3×3 maps. Maps in 2×2 format allow for readability in both full-color and gray scale; 3×3 maps are readable only in color but allow for greater variability in values of displayed variables.

**Table 1:** Examples of Public Health Data Uses for Bivariate Mapping in Cancer Prevention and Control

Variable Combination	Examples	Application
Availability of public health programs to improve prevention or early detection of cancer Population-level use of a cancer screening	Number of Breast and Cervical Cancer Early Detection Program providers within a county relative to potentially program-eligible women County-level estimates of breast and/or cervical cancer use	Identification of geographic areas with low availability of Program providers and low screening rates may reveal important areas to engage additional providers to become involved in the Program.
Accessibility of screening providers Population-level rates of cancer incidence, staging, or mortality	Accessibility of lung cancer screening centers relative to the population of recommended screening age (10) County-level age-adjusted lung cancer mortality	Identification of areas with low access to screening and high disease burden may help communicate to policy makers where public health resources should be targeted and help health systems to identify new potential screening locations.
Accessibility of primary care providers Population-level rates of cancer incidence, staging, or mortality	Accessibility of federally qualified health centers Mortality-to-incidence ratios	Identification with high disease burden relative to low access to primary care services for underserved populations.
Exposure-attributable disease rates Population-level health behaviors	County-level radon-attributable lung cancer mortality rates (7) County-level smoking rates	Identification of areas with particularly high risk of disease due to multiple exposures may be cost-effective targets for public health interventions.
Cancer incidence or mortality in population group #1 Cancer incidence or mortality in population group #2	Breast cancer mortality among non-Hispanic white women Breast cancer mortality among non-Hispanic black women	Identification of particularly stark relative racial disparities may be important geographic targets for states to consider when integrating health equity components into their comprehensive cancer control plans (14).
Cancer screening use Health policy	State-level colorectal cancer screening use (15) State-level Medicaid expansion status (15)	Identify areas where lower rates of screening use may be affected by state-level policy decisions.

## Cervical Cancer Screening Example: Public Health Programing and Surveillance Data

To show how this tool is implemented, we mapped availability of cervical cancer screening providers in South Carolina’s Breast and Cervical Cancer Early Detection Program (BCCEDP) (16) and estimates of ever having had a Pap test among women aged 18 or older from BRFSS at the county level. The BCCEDP program and the BRFSS survey are administered by the state public health department (17). This example uses readily (and often publicly available) data, but we encourage those who implement this method to be sensitive to data use agreements and stipulations for aggregation and scale of geographic scale of data presentation.

**South Carolina’s Breast and Cervical Cancer Early Detection Program.** South Carolina’s BCCEDP provides eligible women with free breast and cervical cancer screening and other services (16). South Carolina women aged younger than 65, who have no insurance or are underinsured, and who meet income requirements are eligible for these services. To be eligible for cervical cancer screening, women must be aged 21 to 64 (18).

**Behavioral Risk Factor Surveillance System.** BRFSS is a telephone-based questionnaire that assesses risk behaviors, chronic health conditions, and use of preventive services (19). The survey is mandated by the Centers for Disease Control and Prevention (CDC) and is administered annually at the state level. Annual surveys include core questions, but states can add optional modules on salient health concerns. BRFSS routinely surveys participants about cervical, breast, and colorectal cancer screening and about cancer-related health behaviors. Optional modules are available on cancer-relevant health care use such as human papilloma virus (HPV) vaccination, lung cancer screening, and cancer survivorship. In our example, we used South Carolina county-level estimates of the percentage of women aged 18 or older who had ever received a Papanicolaou (Pap) test. The state public health department provided these estimates, which were generated by combining responses from 2012, 2014, and 2016 data to maximize sample sizes. However, 3 counties did not have sufficient sample size for stable rates, that is, data were suppressed if the denominator was less than 50 or the 95% confidence interval range was greater than 20%. We also obtained county-level estimates of the percentage of women of recommended screening age (18–65 y) who met the US Preventive Services Task Force screening recommendation for a Pap test (a screening within the past 3 years), but data were suppressed for 9 South Carolina counties (20%), making it an inadequate measure for bivariate mapping (18). Although we used crude estimates in our example, age-adjusted estimates are frequently used by public health professionals and may be useful for bivariate mapping.

## An Overview of Bivariate Choropleth Mapping Creation in ArcGIS

We show how ArcGIS (Esri), a widely used GIS software program, can create bivariate choropleth maps for cancer prevention and control — such as by displaying Pap test use rates and women’s access to cervical cancer screening providers, as we do in our demonstration. Although analysts may use various GIS programs, we used ArcGIS in our example because it is the software used in CDC’s GIS training curriculum for chronic disease (20). In particular, the analyst will implement the following tools in the ArcGIS toolbox and functions in the layer properties and attribute tables: the “spatial join” tools within the ArcGIS toolbox, “symbology” function within layer properties, and “adding and calculating new fields” functions in attribute tables. Additional rendering tools are available to implement bivariate mapping in ArcGIS or other GIS software, but we present our example to develop bivariate maps without additional components (21). Furthermore, we present an example that requires additional, intermediate GIS skills (eg, spatial joins). However, depending on the data that the analyst wants to map, such skills may not be needed to implement this method. The following 7 steps describe the process of creating bivariate choropleth mapping in ArcGIS:

**1. Obtain point and area-level data and shapefiles, (a file format that stores the geometric location and other information about geographic features — polygons/counties, in this case).** The analyst obtains necessary data and shapefiles for implementation. In our example, this includes:

- Address data from the state public health department on BCCEDP cervical cancer screening providers, obtained from the South Carolina BCCEDP manager;
- Area-level (ie, county-level) data on estimates of “ever having had a Pap test” among women aged 18 or older, from the state’s BRFSS coordinator;
- County-level data on the number of women aged 18 to 64 in South Carolina who are uninsured, from the American Community Survey’s Model-Based Small-Area Health Insurance Estimates (SAHIE) for 2017 (22); and
- County-level Topographically Integrated Geographic Encoding and Referencing (TIGER) cartographic boundary shapefiles from the US Census Bureau (23).

**2. Perform data management steps for all data types.** The analyst will perform data management steps for all aforementioned data and shapefiles. In our example, this includes:

- Geocode address data for BCCEDP cervical cancer screening providers that uses the World Geocoding Service (or appropriate address locator file) accessible within ArcGIS, and display these points; and
- Add county-level BRFSS and SAHIE data and TIGER shapefile as layers within ArcGIS, and join area-level data to the shapefile on a linking variable such as Federal Information Processing Standard (FIPS) county codes.

**3. Calculate county-level availability of BCCEDP cervical cancer screening providers. **Geocoded data can be used to determine availability and accessibility of health care services, including service density and area-level travel distance and/or time. We demonstrate the calculation of a service density measure that does not require the use of the spatial analyst tools or the Network Analyst extension. Furthermore, service density measures are more appropriate for larger geographic units, like counties. The analyst will employ the “spatial join” tool within the Analysis ArcGIS tool to sum the number of geocoded points (ie, BCCEDP cervical cancer screening providers) in each county. The target feature will be the TIGER county shapefile, and the join feature will be the geocoded table of points. Within this tool, use a “join one to one” join operation by using the “completely contains” match option. Create a county field and perform a spatial join between the geocoded data and the shapefile. From this joined file, the analyst will add a new field and calculate a new “double” (numeric) variable. This variable will be the summed count of BCCEDP providers by county divided by the count of uninsured women aged 18 to 64 in the county. This age grouping is the closest approximation of BCCEDP eligibility from SAHIE data. We re-scaled this provider-to-population ratio by multiplying by 10,000. This new variable, the second variable of interest, will act as a measure of availability of BCCEDP cervical cancer screening providers relative to a proxy measure of eligibility (women who meet age and insurance status criteria).

**Figure 1:**
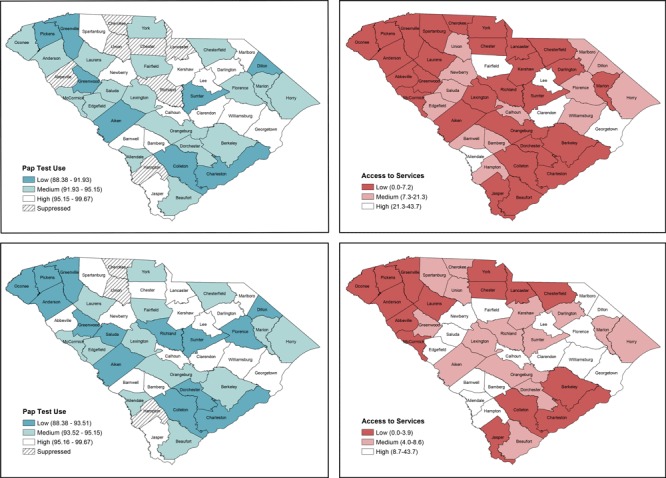
Choropleth maps displaying different classification methods for Papanicolaou (Pap) test use and Breast and Cervical Cancer Early Detection Program (BCCEDP) cervical cancer screening availability. Map A, Pap test use with 3 natural breaks classification; Map B, BCCEDP cervical cancer screening availability using 3 natural breaks classification; Map C, Pap test use using quantile (tertile) classification; Map D, BCCEDP availability using quantile (tertile) classification.

**4. Determine the number of bivariate classes per variable and classification method and create choropleth maps for each variable.** The analyst will explore the distribution of both variables, county-level estimates of “ever having a Pap test,” and county-level availability of BCCEDP providers of cervical cancer screening by using the symbology function under properties for the layer of interest. For this example, 3 counties lack stable rates for Pap test use. These counties should not be considered in the classification scheme and should be selected as a new layer so that they can be displayed as null values, which we display with crosshatches (Figure 1). Recognizing the strengths and limitations of different classification methods (eg, quantile, natural breaks, manual) (Table 2), the analyst should test different classification methods and examine the distribution of values for each variable (24–26). The analyst should consider the number of classes that will be most appropriate for the variables of interest, the number of polygons (ie, counties) displayed, and implications for uses of the resultant bivariate map. For ease in display and interpretation, we recommend that there be no more than 3 classes per variable (ie, 9 total combinations). In our example, we tested 2 classification methods (natural breaks and quantiles) with 3 categories for both variables (Figure 1). Natural breaks classification maximizes the difference between classes while minimizing the differences within classes, but there can be wide variation in the number of counties in each class. Quantile (tertiles in this case) classification ensures that each class has an equal number of counties, but it also means that counties with similar values may be assigned to different categories. However, quantile classification is also more intuitive for lay audiences and can be helpful for indicating the relative disease burden or access scarcity for different areas, which is important when considering the allocation of finite resources. Thus, we chose this classification method for our example.

**Table 2:** Classification Methods for Choropleth Maps

Method	Definition	Strengths	Weaknesses
Natural breaks (Jenks)	Similar values within classes, maximize differences between classes	Explicitly reflects distribution of values Does not arbitrarily place observations with similar values into different groups	Classes do not have equal ranges or equal number of observations Can’t compare across maps
Equal interval	The range of values (the maximum value minus the minimum value) divided into a fixed number of classes	Facilitates comparisons across maps Intuitive Best applied for percentages and temperatures	Not good for skewed data
Quantiles	Equal frequency of values within each n class (eg, tertiles, quartiles)	Ensures each class has an equal number of areas represented Better than the equal interval method for skewed data	Intervals are usually dissimilar in size Similar values may be placed in different classes
*z* score/standard deviation	Values are converted to *z* scores indicating deviation from the mean	Good for comparison of maps (ie, same metric) Useful with normally distributed data	Are not in the units of the values May be less intuitive for lay audiences
Manual	Classes are determined by the analyst (eg, at or above a certain value of interest such as a Healthy People 2020 objective)	Meaningful for audience	Arbitrary Often not statistically driven
Classless	Continuous values varying by intensity of color shading	Shows variation more accurately	May be difficult to distinguish values across large areas

**5. Categorize variable combinations accordingly.** For each variable (in this example, Pap test use and BCCEDP availability), create a new “tertile” variable for each variable in the attribute table, representing low, medium, and high values. This can be achieved by, for example, “selecting by attribute” for the variable of interest and selecting all values in the lowest tertile. When counties with these values are selected, use the “calculate” function to assign the tertile group for the new variable. Repeat this for each tertile grouping for both new variables. The new variable should now have 3 values reflected in each: 1, 2, 3 or low, medium, high, whichever naming scheme is more intuitive. From these 2 new variables, create a third new variable within the attribute table again by using the “select by attribute” function to create 9 values based on the combination of values from the 2 new tertile groups:

1. High access/high use
2. High access/medium use
3. High access/low use
4. Medium access/high use
5. Medium access/medium use
6. Medium access/low use
7. Low access/high use
8. Low access/medium use
9. Low access/low use

In total, we created the following county-level variables in steps 3 to 5: 1) availability of BCCEDP cervical cancer screening providers (step 3); 2) BRFSS Pap test use tertiles (step 5); 3) BCCEDP availability tertiles (step 5); and BRFSS Pap test and BCCEDP tertile overlap (step 5).

**Figure 2:**
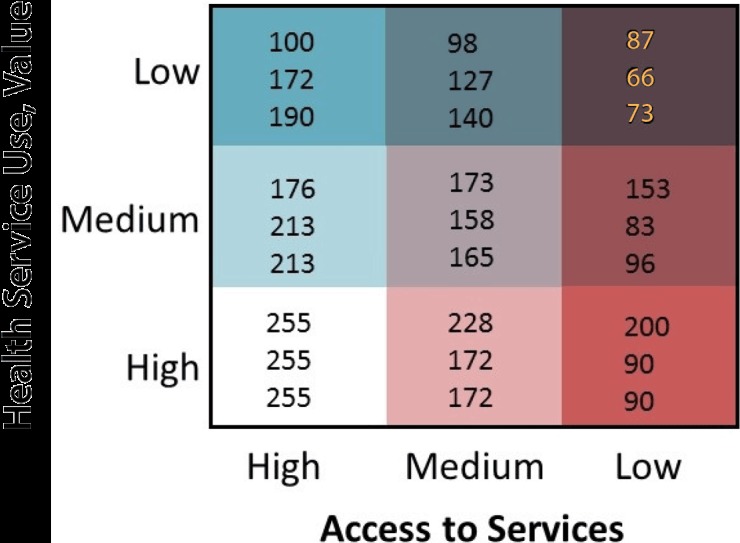
Sample 3×3 bivariate map legend displaying visually distinguishable color scheme with red, green, blue color (RGB) codes displayed.

**Figure 3:**
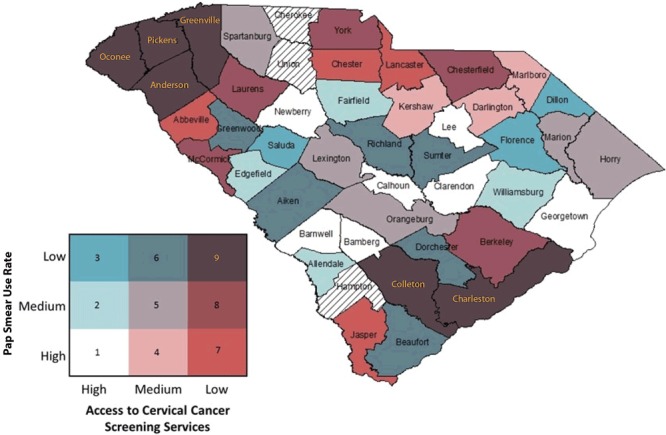
Bivariate map displaying Pap test use and Breast and Cervical Cancer Early Detection Program cervical cancer screening availability with color-coded legend. (Counties with hatch marks had insufficient data to map.)

**6. Assign a visually distinguishable color scheme and legend for a bivariate map.** Because nine colors will be displayed, it is important that the hues symbolizing the values of the new bivariate variable be visually distinguishable. We suggest that analysts use the Color Brewer website (27) or refer to suggested color schemes from Joshua Stevens (28). We provide an example of a color palette and legend in Figure 2. This figure is overlaid with the red, green, blue (RGB) color codes for each respective color displayed to allow the analyst to modify the fill colors for their maps within the symbol selector function. We created our legend in Microsoft PowerPoint, but an .mxd file is available for download to create a bivariate legend (21). In displaying these values, we suggest that the analyst designate the grouping with the worse outcome or access in the darkest color hue. In our example, this will be the counties with the lowest BCCEDP availability and the lowest Pap test use rate. For maps that display disease burden, the counties with the highest values should be displayed in the darkest color hue. We also recommend that users review best practices for mapping cancer data by including relevant elements: title, legend, labels, and other information as needed (5). Best practices include development of a descriptive title, a clear legend, and helpful geographic and data labels (eg, county names). The final map, for example, is displayed in Figure 3.

7. **Interpret bivariate map for cancer prevention and control application.** After the bivariate map has been developed with the appropriate elements, analysts can interpret the map. In our example map (Figure 3), we identified that counties (Oconee, Pickens, Greenville, and Anderson) in the northwestern-most part of the state and Colleton and Charleston counties in the southeastern-most part of the state fell into the low-access/low-use group, suggesting that these counties may be important targets for increased resource allocation or physician engagement to improve the number of BCCEDP providers relative to the population in need of such services. Additionally, counties with high access but low use (Saluda, Florence, and Dillon) may be important targets for educational interventions, because services are available but are not being used sufficiently. Such information may be helpful for state public health departments to help allocate resources and implement interventions, and for nonprofit organizations as they advocate for increased resources. Of note, 3 counties had null values because they had insufficient data on Pap test use.

## Strengths and Limitations of Bivariate Mapping for Cancer Prevention and Control

The strength of the GIS mapping approach is that it is easy to implement for users with an intermediate GIS skill set and is ideal for the display of sub-state variables that are often readily available from federal or state public health surveillance systems and programs. Furthermore, it allows for the display of 2 variables simultaneously using a single-color scheme. Additionally, the resultant bivariate map provides useful information for public health, nonprofit groups, and other stakeholders who want to identify geographic targets for resource allocation decisions and intervention planning. In our experience, geographic mapping is received positively in both lay and scientific communities, especially over typical numerical presentations (tables of rates or percentages). It allows the audience to quickly and easily identify problem areas. However, this method is not without its limitations. First, although users may implement the classification method most appropriate for their data and map use, each method has its strengths and weaknesses, which users will have to weigh when creating such maps. Second, display of county-level surveillance data often yields suppressed data because of unstable rates in sparsely populated rural counties. Thus, some counties may not have sufficient data for bivariate mapping, as in Figure 3. However, this problem can be mitigated through implementation of rate smoothing or small-area estimation approaches if the analyst has access to nonaggregated data and appropriate statistical training (29–31). Similarly, displaying spatial access to care by using a service density approach, as we do in this example, has limitations. This approach does not indicate where screening providers are located within a county. It also assumes people have access to or seek care only within their county. More rigorous GIS approaches that consider distance to health care services and seeking health care across county boundaries are typically implemented at smaller geographic scales (zip codes or census tracts), for which it is difficult to provide area-level rates or estimates of health care use or disease burden.

## Conclusions

Implementing bivariate mapping approaches to simultaneously display 2 relevant variables is an effective, but underused method to inform cancer prevention and control efforts. Applied to cancer control planning, this method can display surveillance data on risk factors, screening, incidence, and mortality and data on socioeconomic factors or availability and accessibility of health care resources. GIS users can implement a straightforward set of data management and symbology steps in ArcGIS to develop bivariate maps. The resultant maps can be interpreted to inform allocation of resources, geographic targeting of interventions, and advocacy efforts to inform cancer prevention and control efforts.
